# Applications of a novel reciprocating positive displacement pump in the simulation of pulsatile arterial blood flow

**DOI:** 10.1371/journal.pone.0270780

**Published:** 2022-12-13

**Authors:** Adam Menkara, Ahmad Faryami, Daniel Viar, Carolyn Harris

**Affiliations:** 1 Department of Biomedical Engineering, Wayne State University, Detroit, Michigan, United States of America; 2 Department of Computer Science and Engineering, University of Toledo, Toledo, Ohio, United States of America; 3 Department of Chemical Engineering and Materials Science, Wayne State University, Detroit, Michigan, United States of America; Khalifa University of Science and Technology, UNITED ARAB EMIRATES

## Abstract

Pulsatile arterial blood flow plays an important role in vascular system mechanobiology, especially in the study of mechanisms of pathology. Limitations in cost, time, sample size, and control across current in-vitro and in-vivo methods limit future exploration of novel treatments. Presented is the verification of a novel reciprocating positive displacement pump aimed at resolving these issues through the simulation of human ocular, human fingertip and skin surface, human cerebral, and rodent spleen organ systems. A range of pulsatile amplitudes, frequencies, and flow rates were simulated using pumps made of 3D printed parts incorporating a tubing system, check valve and proprietary software. Volumetric analysis of 430 total readings across a flow range of 0.025ml/min to 16ml/min determined that the pump had a mean absolute error and mean relative error of 0.041 ml/min and 1.385%, respectively. Linear regression analysis compared to expected flow rate across the full flow range yielded an R^2^ of 0.9996. Waveform analysis indicated that the pump could recreate accurate beat frequency for flow ranges above 0.06ml/min at 70BPM. The verification of accurate pump output opens avenues for the development of novel long-term in-vitro benchtop models capable of looking at fluid flow scenarios previously unfeasible, including low volume-high shear rate pulsatile flow.

## Introduction

Pulsatile arterial blood flow has been shown to have inherent properties that are integral to the normal function of specific organ and tissue systems [1, 2]. The ability to generate pulsatile flow is important in the development of in-vitro models to simulate real-life conditions. However, many modern fluidic devices such as peristaltic pumps resort to utilizing constant or harmonic-based fluid flow. This is especially true in low volume conditions due to limitations in producing physiologically accurate low volume flow [3].

While extremely useful when studying systemic impact, animal studies are limited in their ability to directly and simultaneously mimic human physiologic arterial blood flow. These studies are also usually very time-intensive to setup and are limited in the range of physiologic and pathophysiologic scenarios that can be measured and tested dynamically. In-vitro models maintain control and dynamic recording potential. However, significant cost and time investment required to build, verify, and validate a test setup prior to conducting flow based biomechanistic in-vitro studies limits their application.

The most commonly used example of an in-vitro modelling positive displacement-based fluid producing device would be a peristaltic pump. Individual lab grade peristaltic pumps can range from $50USD to $200USD, with the corresponding tubing ranging from $20 to $100 per meter based on flow range capabilities for a single channel. In order to gain an accurate assessment of system variables, In-vitro modelling setups often require the use of a minimum of 5 channels per testing scenario. This brings the minimum buy-in cost to $350 with the assumed use of 1 meter of tubing per channel. However, this price often reaches well over the upper limit of the price ranges ($1000+/channel) as the lower flow rates require more expensive pumps and tubing per channel [4]. Peristaltic pumps and similar in-vitro modelling pumps are also relatively time consuming to setup. Because of the complex tubing attachment mechanisms, as well as the large size of pump for each channel, a complex setup must be devised beforehand with careful consideration for pump and modelling chamber placement [5].

In our previous study, the accuracy of the reciprocating positive displacement pump was demonstrated across a range of 0.01ml/min to 0.7ml/min with an R^2^ value of 0.9998 [6]. The pump was originally produced in order to simulate the high shear rate pulsatile waveform of cerebral spinal fluid for in-vitro modelling scenarios. Other studies have shown that high shear rate pulsatile blood flow have aided in the proliferation of cells under in-vitro modelling scenarios, as well as abnormalities in blood shear rate being an indicator of vascular disease [7–9]. These are only some of the scenarios that could be modelled in lab with the use of a pump capable of recreating high shear pulsatile flow through a range of scenarios. The lack of a versatile pump capable of replicating this pattern of flow in a laboratory setting with high accuracy was the direct motivation for the application of the pump in this manner. To expand the verified flow rate range and investigate the capabilities of the pump in novel applications of arterial blood flow in organ systems, a literature search was conducted collecting blood flow rate data from organ systems and was simulated using the pump spanning three ranges; low (0ml/min– 0.4ml/min), mid (0.4ml/min– 1.5ml/min) and high (3ml/min– 16ml/min) bulk flow rates.

This study presents the testing and verification of a novel positive displacement pump and operating program through the simulation of literature-based blood flow data of organ systems spanning human ocular, human fingertip and skin surface, human cerebral, and rodent spleen. This pump aims to, unlike current state of the art pumps, to generate a wide range of high shear rate, pulsatile bulk flow rates while resolving issues of setup time and cost which feature in previous in-vitro modelling flow generating pumps. This is achieved through the use of a syringe-based positive displacement method of driving fluid flow in conjunction with the precise control of programmable input variables through a user interface. These allow for the generation of a wide range of pulsatile low volume fluid flow rates and waveforms, while keeping a low total unit cost of under $200, with cost per channel being under $40. Furthermore, the incorporation of a robust check valve system enables the reciprocating action of the pump, allowing it to retract back to its original starting position once the syringes have been compressed fully within the pump to refill the syringes with the selected fluid. This valve system then fully automates the pump for long term fluid flow experiments.

In order to verify the accuracy of output bulk flow rate and pulsatile amplitude and frequency, the output of the pump will be measured and compared to the expected outcome under the scenarios described previously.

## Materials and methods

The testing and verification of the novel reciprocating positive displacement pump involves the simulation of literature-based blood flow data from organ systems spanning human ocular, human fingertip and skin surface, human cerebral, and rodent spleen. These blood flow data span arbitrarily defined low (0ml/min– 0.4ml/min), mid (0.4ml/min– 1.3ml/min) and high (3ml/min– 16ml/min) flow rate ranges aimed to provide flow rate performance for different flow applications. Simulations of each of the individually collected testing scenarios will be performed and measurements of output bulk flow rate and waveform will be collected. A regression analysis will be performed comparing the expected and output bulk flow rates of the pump.

### Reciprocating positive displacement pump

**Fig 1:**
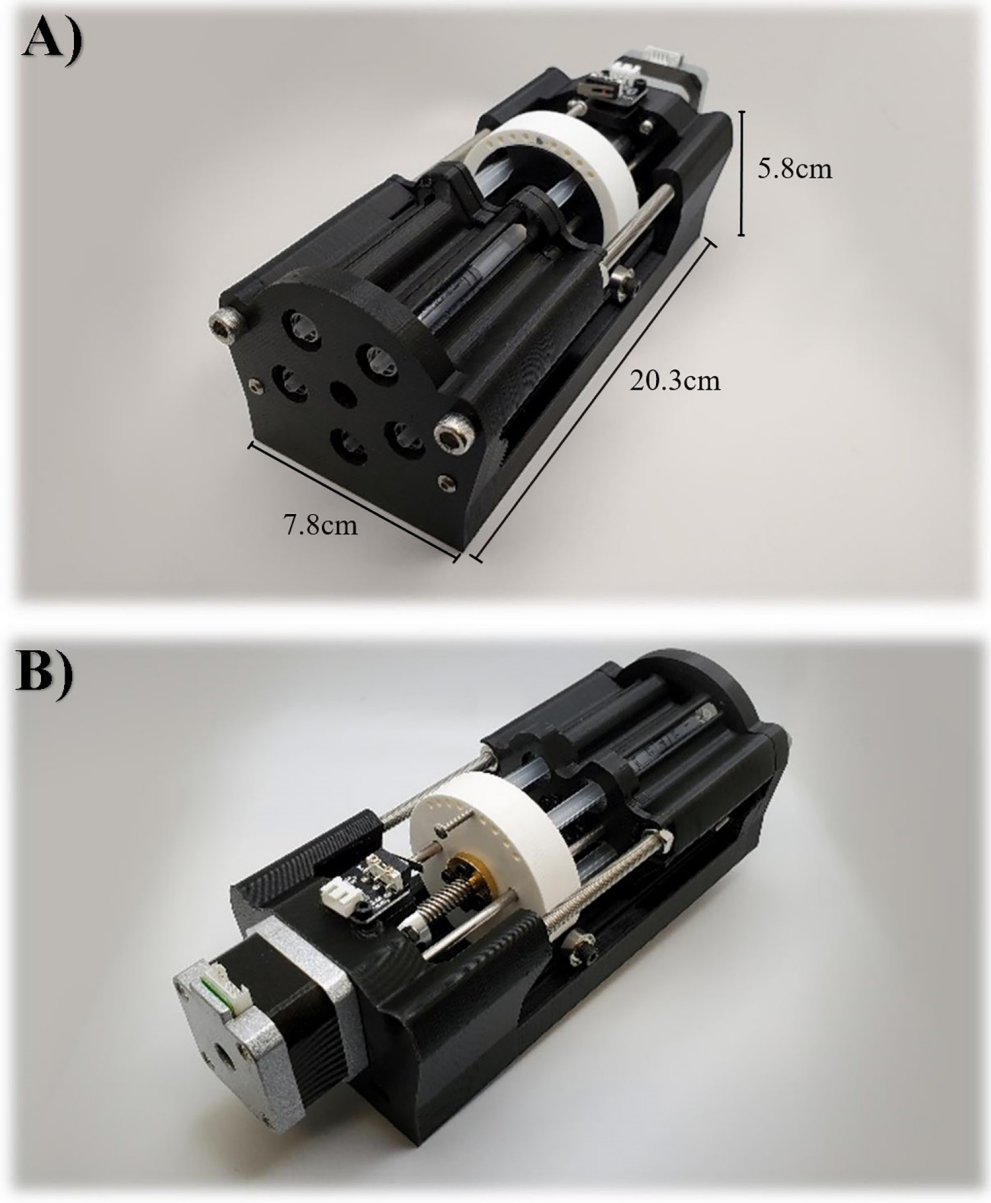
**Reciprocating positive displacement pump.** Front view with scale bars (a) and rear view (b) of fully assembled reciprocating positive displacement pump.

The chassis of each reciprocating positive displacement pump is comprised of 3D printed parts made of polylactic acid plastic (PLA) manufactured using an Anycubic I3 Mega printer (Anycubic Technology CO., Limited, HongKong) [6]. The design consists of five 3ml syringes per pump, allowing flow output from five total channels per pump (Fig 1). A stepper motor, linear bearings, motor coupler, and lead screw assembled in line with each channel allow for the conversion of rotational motion into linear movement. The total cost of components is under $200, with variations of the price based on situational part sourcing. This puts the initial cost per channel to be under $40.

Using Python, we developed a proprietary software allowing for the direct input through a simple user interface of user profile variables consisting of output bulk volume rate, pulsation frequency, and pulsation amplitude. The following open-source packages used in the development of the software include Tkinter, OpenCV, and Printrun [10–12]. Based on user input per profile, rotational speed and duration were calculated by the software. Mechanical inefficiencies of the pump and other components were accounted for through the program. This information is then sent to an Arduino-based Creality 3D v1.1.4 board which is then interpreted and used to drive stepper motor movement. Each Creality 3D v1.1.4 board is capable of simultaneously running three pumps, a total of 15 channels per board. An automatic sterilization function was also built within the program consisting of a constant reciprocating action of the pump for a total of 20 minutes, cycling 99% isopropyl alcohol throughout the duration of the cleaning function. This is followed by three cycles of deionized water to expel any remaining isopropyl alcohol from the tubing system.

Once the syringes have reached their fully compressed state, the program then automatically resets the pump back to its original position, extending the syringes fully. The use of individual check valves immediately after the output from the syringes enables this retracting motion of the pump to automatically refill the syringes. This retraction time takes a total of approximately eight seconds. Each check valve limits the chances of cross-contamination between sample outputs and between input and output within each channel. Luer locking fittings used by the check valves and syringes allowed for simple and robust fluidic connections between all components. Input and output for each individual channel consisted of 50cm in length and 3mm inner diameter silicone tubing into their respective check valves.

### Systolic time interval

An important variable in the accuracy of individual pulsations is systolic time interval. An analogous representation of ventricular systolic time interval is created through the implementation of the systole time user input variable. This allows for direct control over beat cycle length independent of volume rate or frequency. Ideally, beat cycle duration can be approximately equal to two times the input systole time, although external variables such as system compliance may affect this.

### Amplitude

Manipulation of amplitude of individual pulsations is based on the multivariable user input per profile. Resulting amplitude is directly correlated to volume rate and compliance of the tubing and flow system, and inversely correlated to both systole time and beat frequency. The dampening factor is a representation of compliance of the entire tubing system, and refers to the tubing system itself, attached chambers to the tubing and fluid viscosity. All these variables such as tubing diameter can be manipulated to better fit the intended modelling parameters. The relationship between these variables and their effects on amplitude can be seen in Eq 1:

$$\mathrm{Amplitude}\left(\frac{\mathrm{ml}}{\min}\right)=\frac{\mathrm{Volumerate}(\frac{\mathrm{ml}}{\min})\cdot \frac{60\mathrm{seconds}}{\min}}{\mathrm{Systoletime}(\mathrm{seconds})\cdot \mathrm{Heartrate}(\frac{\mathrm{beats}}{\min})}\cdot \mathrm{Dampening}\;\mathrm{Factor}$$

The incorporation of a method of simply and directly manipulating pulsatile amplitude allows for the freedom to explore domains of clinical applications previously unexplored.

### Testing parameters

The organ systems used to populate these arterial blood flow ranges are human ocular, human fingertip, human cerebral, and rodent spleen (Table 1). Unless otherwise noted, an input of 70BPM and 0.1 second systolic time were used for all human simulations and 380BPM and 0.054 second systolic time for all rat-based simulations [13–15]. While any heartrate within physiologic domain could have been selected, an average resting 70BPM for humans and 380BPM for rats was chosen unless otherwise specified. Systolic time was not provided in any study simulated throughout the flow ranges, therefore requiring the use of arbitrary systolic times of 0.1 seconds for human and 0.054 seconds for rat-based simulations.

**Table 1:** Organ systems scenarios and corresponding flow rate, beat rate, and systolic time input profiles into reciprocating positive displacement pump.

Organ System	Measuring Device	Condition	Flow Rate (ml/min)		Beat Rate (BPM)	Systolic Time (s)	Reference
			Avg.	Std. Dev			
(F)	(PP)	post-caffeine	0.029	0.004	71	0.1	[16]
(R)	(LD)	baseline	0.033		70	0.1	[17, 18]
(F)	(VOP)	venous (lower)	0.056		70	0.1	[19]
(F)	(PP)	pre-caffeine	0.067	0.009	74	0.1	[16]
(R)	(LD)	baseline	0.08	0.012	70	0.1	[20]
(F)	(PP)	baseline	0.088	0.015	76	0.1	[16]
(F)	(VOP)	venous (critical)	0.2		70	0.1	[19]
(R)	(PC)	baseline	0.261	0.087	70	0.1	[21]
(F)	(VOP)	venous (upper)	0.42		70	0.1	[19]
(O)	MRI	(lower)	0.444		70	0.1	[21]
(S)	(TTP)	(pre-caudal (Li))	0.6	0.1	380	0.054	[22]
(S)	(TTP)	(pre-rostral (Li))	0.8	0.3	380	0.054	[22]
(O)	MRI	(upper)	0.803		70	0.1	[21]
(C)	MRI	baseline	0.917	0.281	70	0.1	[21]
(O)	(LP)	baseline	1		70	0.1	[23, 24]
(S)	(TTP)	(post-caudal (Li))	1.0	0.2	380	0.054	[22]
(S)	(TTP)	(post-rostral (Li))	1.2	0.1	380	0.054	[22]
(MA)	(PC)	baseline	6	3	70	0.1	[25]
(OA)	(PC)	baseline	11	5	70	0.1	[25]

(F)–Fingertip, (R)–Retinal, (O)–Ocular, (C)–Choroidal, (S)–Splenic arteriovenous in rats, (LP)–Langham Pneumotonometer, (Li)–Ligation, Middle Meningeal Artery—(MA), Ophthalmic Artery—(OA), Venous occlusion plethysmography—(VOP), Laser doppler—(LD), Phase contrast MRI (PC), Pulse plethysmography (PP), Transition time probes (TTP)

**Fig 2:**
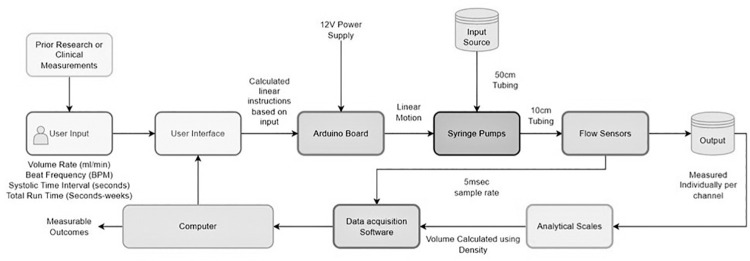
Schematic diagram of testing setup. Illustrates flow of fluids and data collection locations of user input verification.

These systolic times are used as a representation of true systolic time, however changes in systolic times can be made for direct manipulation of pulsatile amplitude independent of flow volume rate and beat rate. Degassed, deionized water, measured and verified at 1 gram/milliliter of water at room temperature, was used to measure the accuracy and precision of pump output. A total of 10, ten-minute weight measurements were performed across 10 pump channels using the pump and tubing setup schematic seen in Fig 2. per input user profile. The sterilization function described earlier was used to properly clean and prime pumps before each testing session. Individual beakers were used per channel, weighed after the time period of each test was completed. Weight measurements were conducted using a Mettler Toledo AT261 DeltaRange Analytical Balance (Mettler-Toledo, LLC, USA).

Each individual profile tested also had flow data obtained from a flow sensor placed at the immediate output of a channel check valve for each individual profile tested. The flow sensor used to collect flow data was a Sensirion SLF06 series flow sensor (Sensirion AG, Switzerland). Flow data was used to determine consistency of amplitude and volume rate within the same test, accurate beat rate throughout the duration of each test, and differences of volume rate and amplitude across different profiles.

### Statistical analysis

IBM SPSS Statistics (IBM, USA) was utilized to perform a linear regression to analyze the measured output of the pumps to the expected value over the 0.025 mL/min to 16 mL/min range. A linear regression was also performed on the output low, mid, and high bulk flow rate ranges individually. Individual plots were created for the low, mid, and high flow rate ranges to better assess the fit of the data to the linear regression, and the subsequent R^2^ value. A linear regression was chosen because of the linearity of the expected and actual output data from the pump, and because the prevalence of an individual data point having no bearing on the actual accuracy of the pump. The y-intercept of output equations from the linear regressions were then used to further assess the accuracy of the pump, with a percent difference generated from the optimal y-intercept value for 0 (zero). An ANOVA test was also performed on the linear regression of the full range of expected output pump bulk flow rates, with the intention of assessing the validity of the linear regression itself as a tool for measuring pump accuracy. Bland-Altman plots were also generated to better assess and describe the levels of absolute flow rate error within each of the pump ranges. Absolute and relative standard deviations were generated from the 10 measurements made for each of the scenarios performed from Table 1. These were created to better indicate the consistency of the pump within an individual scenario.

## Results

A summary of the total verified range of the pump for amplitude, beat rate, and bulk volume rate is shown in Table 2.

**Table 2:** Verified pulsation rate, volume rate and amplitude range achievable by the reciprocating positive displacement pump.

Category	Minimum	Maximum
Pulsation Rate $\left(\frac{Beats}{min}\right)$	1	400
Output Volume Rate $\left(\frac{ml}{min}\right)$	0.01	16
Amplitude ($\frac{ml}{min}$)	0.02	65

### Volumetric analysis

**Fig 3:**
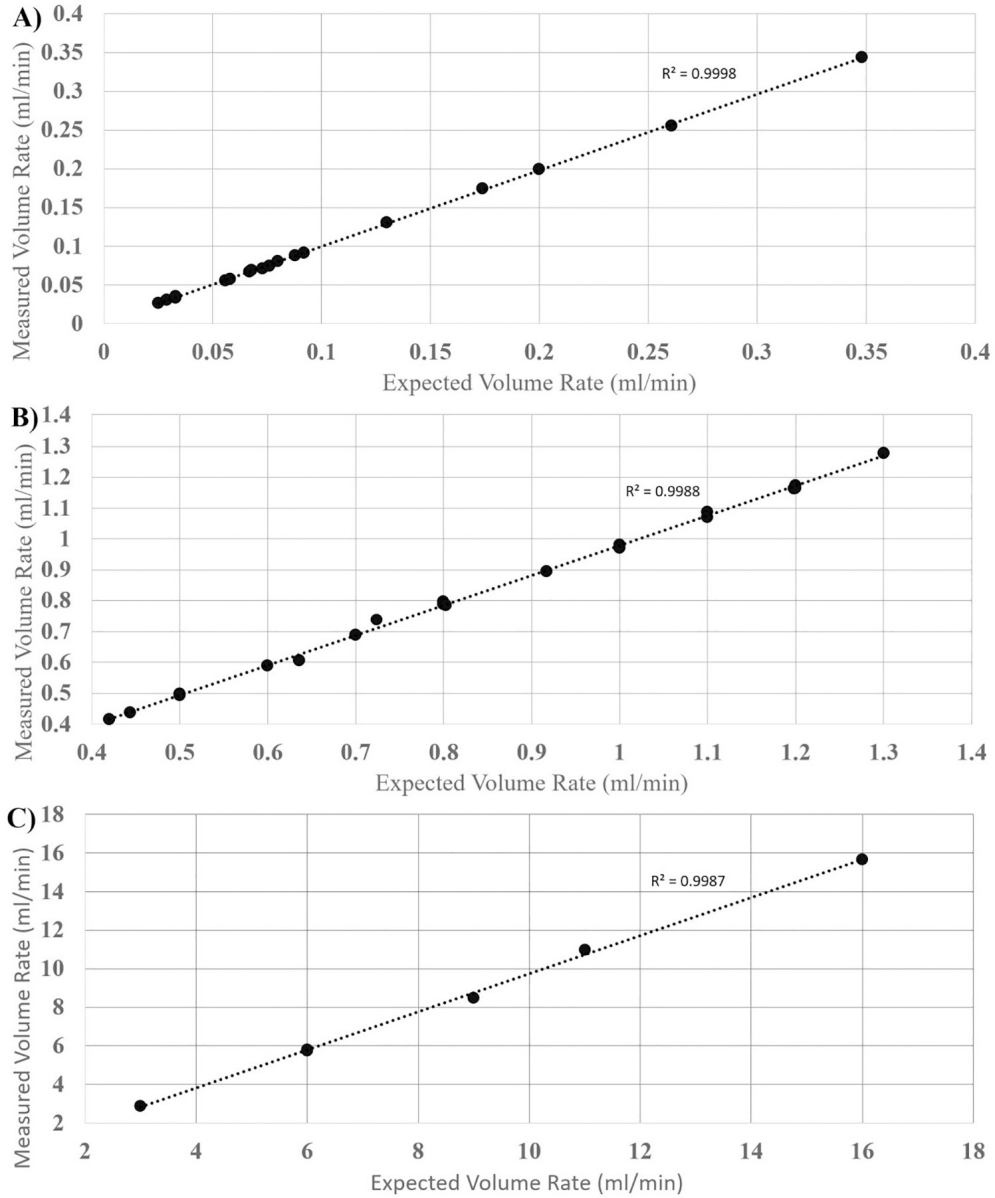
Expected vs. measured low, mid, and high bulk flow rate ranges. Illustrates the expected versus mean of measured volume rate across 10 channels for low flow ranges (0ml/min– 0.4ml/min) (A), mid flow ranges (0.4ml/min– 1.4ml/min) (B), and high flow ranges (3ml/min– 16ml/min) (C) with a linear function showing a regression and R^2^ value for each plot.

The pumps simulated the volumetric flow rates for the low, mid, and high bulk flow rate ranges spanning a total range of 0.025ml/min to 16ml/min for the organ systems of the human eye, human fingertip and skin surface, rat spleen and human cerebral blood flow rates (Fig 3, Table 3).

**Table 3:** Verified organ systems explored in this paper within achievable range of reciprocating positive displacement pump.

Species	Simulated Organ System	Minimum Input Flow Rate ($\frac{ml}{min}$)	Minimum Simulated Output by Pump $\left(\frac{ml}{min}\right)$	Maximum Input Flow Rate ($\frac{ml}{min}$)	Maximum Simulated Output by Pump $\left(\frac{ml}{min}\right)$
Human	Retina	0.033	0.0351856	0.348	0.3439348
Human	Ocular Choroidal	0.636	0.6050832	1.198	1.16184841
Human	Total Pulsatile Ocular	0.444	0.4363839	1	0.9804422
Human	Fingertip	0.025	0.0260722	0.42	0.4150397
Human	Ophthalmic Artery	6	5.8181232	16	15.6552252
Human	Middle Meningeal Artery	3	2.8941562	9	8.5042032
Rat	Splenic Arteriovenous Flow Differential	0.5	0.4931776	1.3	1.2774671

**Fig 4:**
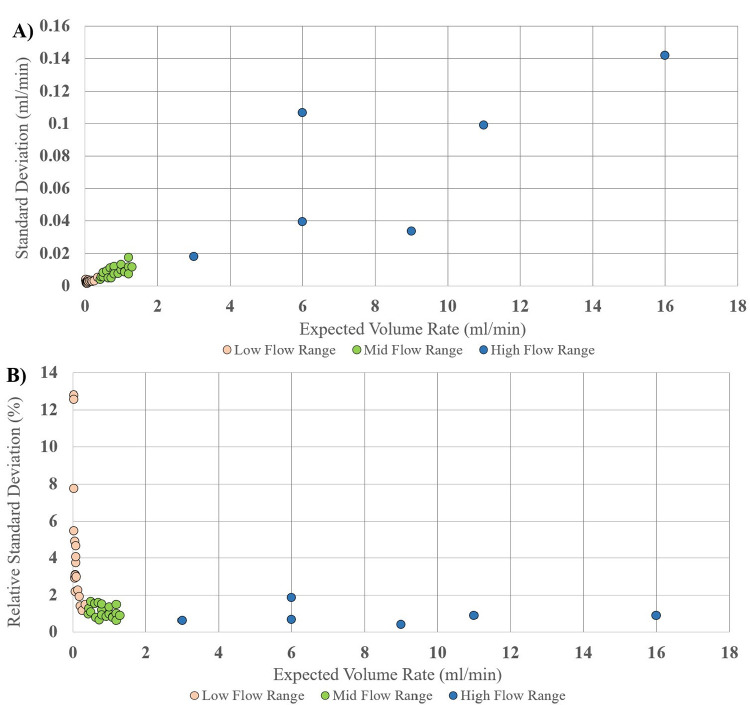
Standard deviation across full bulk flow rate range. Illustrates the standard deviation(A) and relative standard deviation(B) across full range of tested flow ranges.

**Fig 5:**
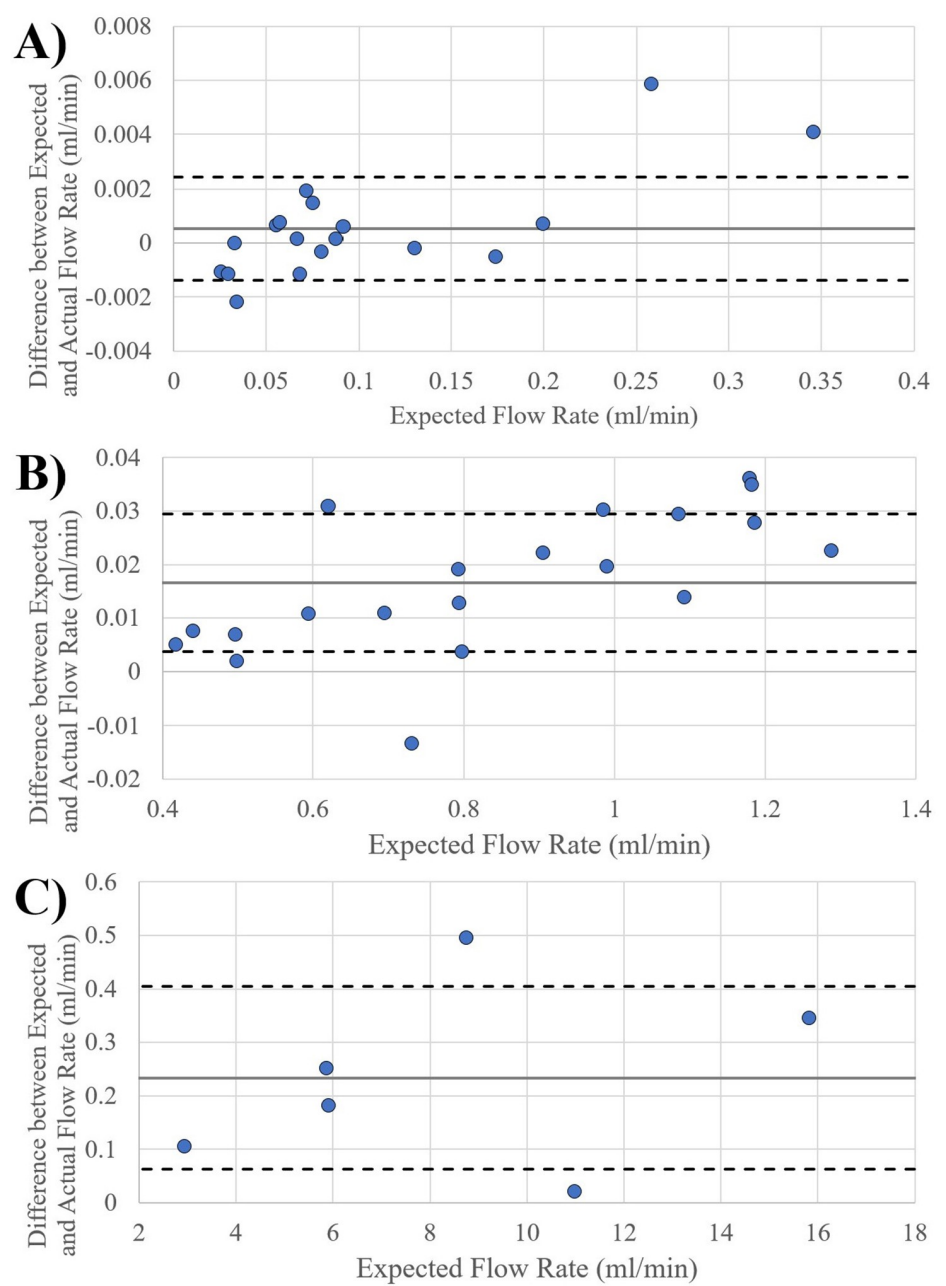
Bland-Altman plot across bulk flow rate ranges. Illustrates Bland-Altman plot across low(A), mid (B), and high (C) bulk flow rate ranges.

Across the entire range of flow rates, a total of 430 ten-minute weight measurements taken from the pumps had a mean absolute error and mean relative error of 0.040854 ml/min and 1.385164% respectively. The linear regression of the low, mid, and high flow rate ranges (Fig 3A–3C, respectively) tested yielded R^2^ values 0.9998, 0.9988 and 0.9987, respectively. The subsequent linear regression equations yielded y-intercept values of 0.0012, 0.0096, and -0.1353 respectively, with the optimum being 0. The absolute difference compared to the optimum y-intercept for the respective flow rate ranges are 0.12%, 0.96%, and 1.353%. The R^2^ value across the entire range of flow rates yielded a value 0.9996, with a y-intercept of -0.0014, yielding an absolute difference in optimum y-intercept of 0.14. The ANOVA performed on the linear regression of the entire range of flow rates yielded a p value of less than 0.01. The standard deviation across the total range of 0.025ml/min to 16ml/min was 0.00151ml/min to 0.14196ml/min presented in Fig 4(A). Relative standard deviation indicates the pumps’ precision and consistency for individual tests across the range of tested flow rates (Fig 4(B)). Additionally, a Bland-Altman plot was generated for added clarity in displaying the standard deviation shift across individual low, mid, and high flow rate ranges (Fig 5).

Although there is a proportional change in the absolute standard deviation in flow rate, the relative standard deviation remains relatively constant throughout the entire tested flow rate range, with the exception of a significant increase between the 0–0.5ml/min expected volume rate ranges.

### Pulsatile flow

**Fig 6:**
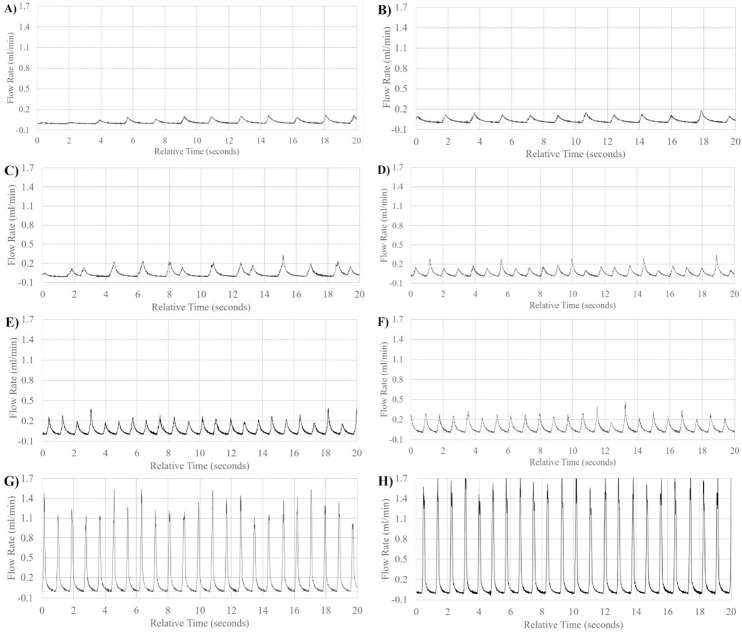
Low flow range pulsatile waveform patterns. Visual representation of flow waveform pattern of mean flow in order from lowest to highest bulk volume rate simulated in low flow rate range; effects of caffeine on fingertip blood flow autoregulation (post-caffeine) (A), retinal blood flow by laser doppler velocimetry (B), fingertip blood flow by venous occlusion plethysmography (C), effects of caffeine on fingertip blood flow autoregulation (pre-caffeine) (D), retinal blood flow by laser doppler velocimetry (E), effects of caffeine on fingertip blood flow autoregulation (baseline) (F), critical vasoconstriction temperature for fingertip blood flow (G), retinal blood flow by phase contrast MRI (H).

**Fig 7:**
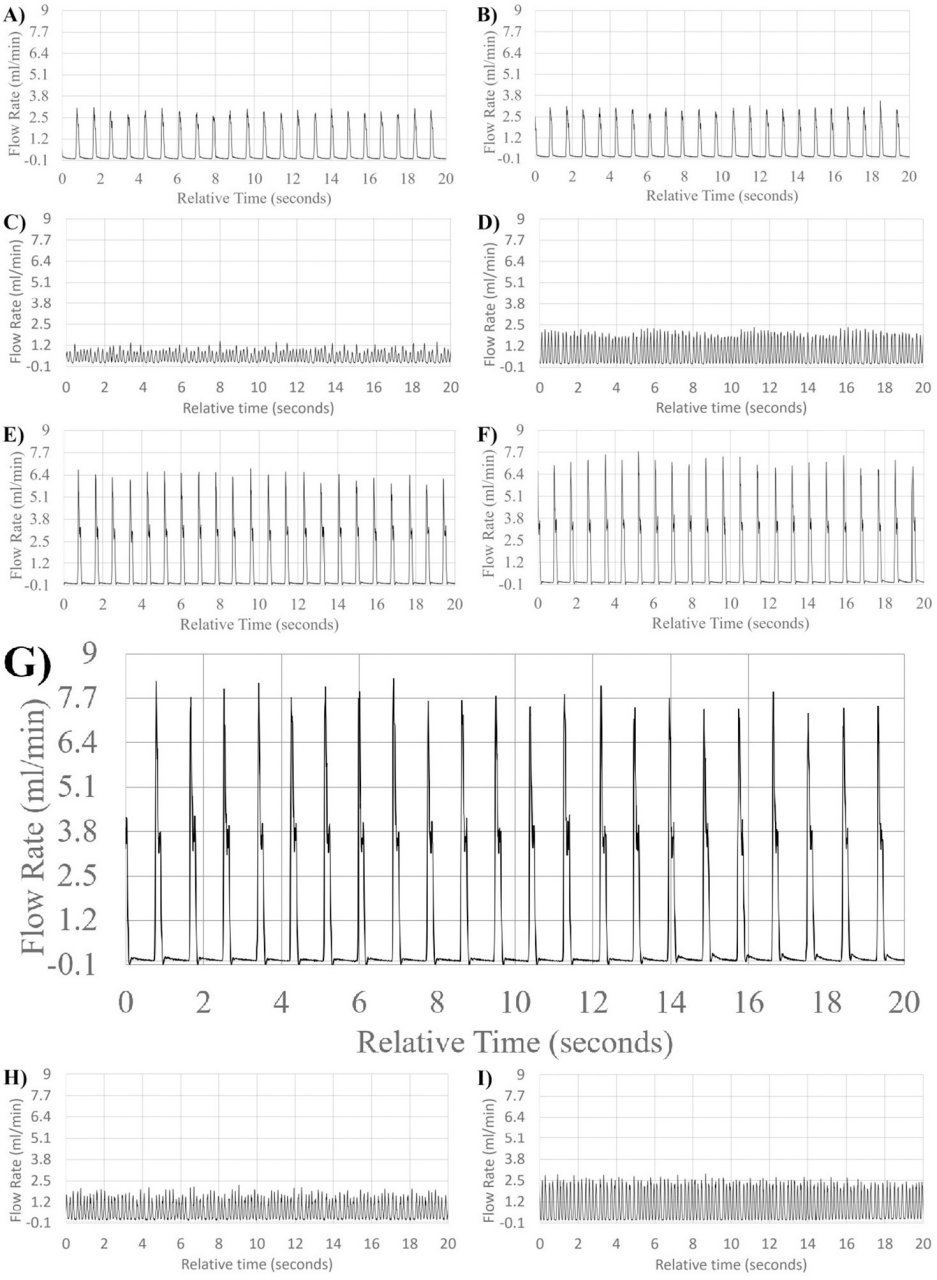
Mid flow range pulsatile waveform patterns. Visual representation of flow waveform pattern of mean flow in order from lowest to highest bulk volume rate simulated in mid flow rate range; fingertip blood flow by venous occlusion plethysmography (A), total pulsatile ocular blood flow by phase contrast MRI (B), splenic arteriovenous flow differential in rats (pre-caudal ligation) (C), splenic arteriovenous flow differential in rats (pre-rostral ligation) (D), total pulsatile ocular blood flow by phase contrast MRI (E), ocular choroidal blood flow by phase contrast MRI (F), total pulsatile ocular blood flow by Langham pneumotonometer (G), splenic arteriovenous flow differential in rats (post-caudal ligation) (H), splenic arteriovenous flow differential in rats (post-rostral ligation) (I).

**Fig 8:**
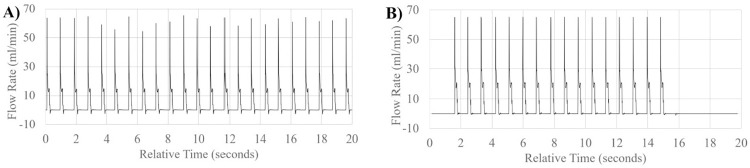
High flow range pulsatile waveform patterns. Visual representation of flow waveform pattern of mean flow in order from lowest to highest bulk volume rate simulated in high flow rate range; Middle meningeal Artery (A), Ophthalmic Artery (B).

Flow measurements from the pump illustrated accurate pulsatile flow according to beat rate and volume rate in humans and rats (Figs 6–8). Flow rates under 0.06ml/min with an input of 70BPM were too small and were unable to simulate accurate 70BPM pulsatile waveform flow. Therefore, waveforms presented in Fig 6A–6C do not have the correct beat counts in the relative time frames, although the pump remains volumetrically correct due to inbuilt catchup functions within the proprietary program. However, A total of ~23 total beats were counted across the 20 seconds displayed for all other displayed flow patterns, equating to the input value of ~70BPM.

**Fig 9:**
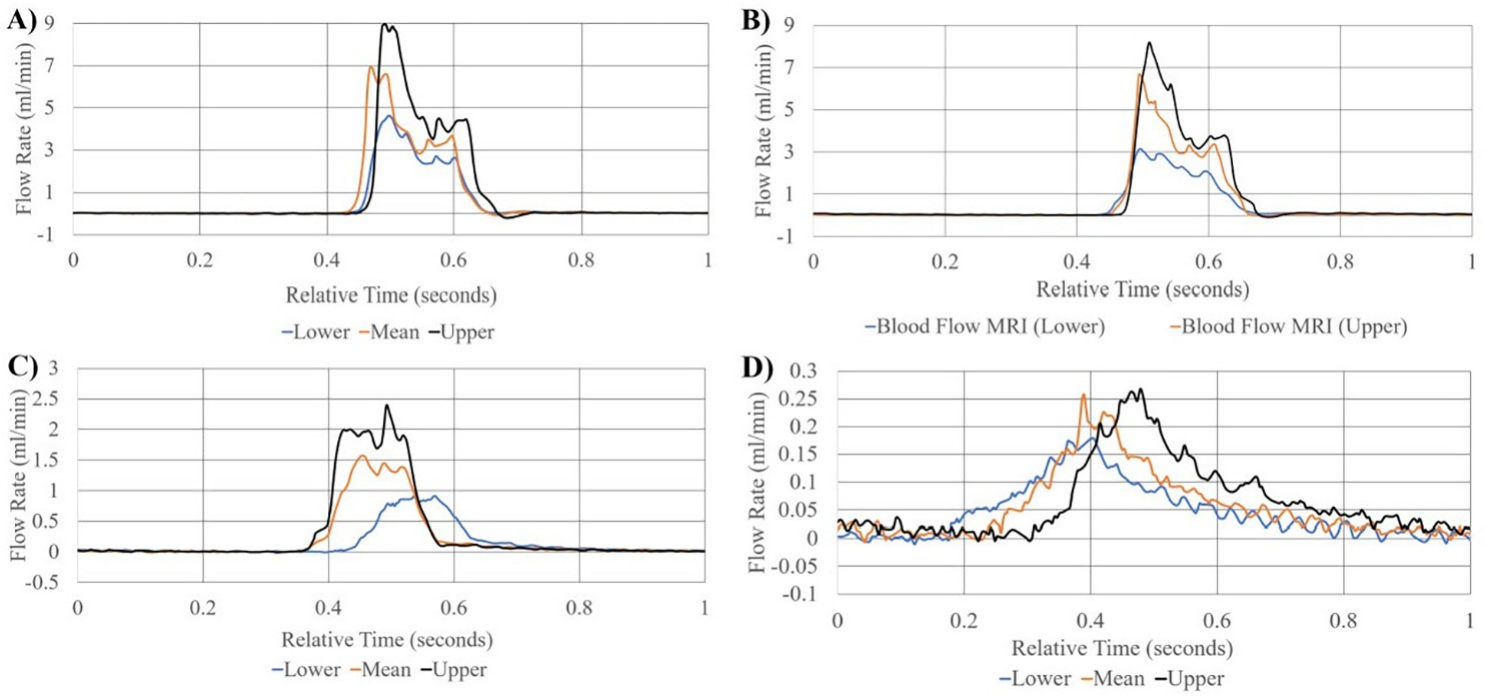
Visualization of single second pulsatile waveform patterns. Visual Representation of comparison of lower, mean, and upper limits of flow waveform patterns across 1 second for choroidal blood flow rates (A), total pulsatile ocular blood flow measurements (B), retinal blood flow gathered through MRI (C), retinal blood flow gathered using Laser Doppler velocimetry (D).

**Fig 10:**
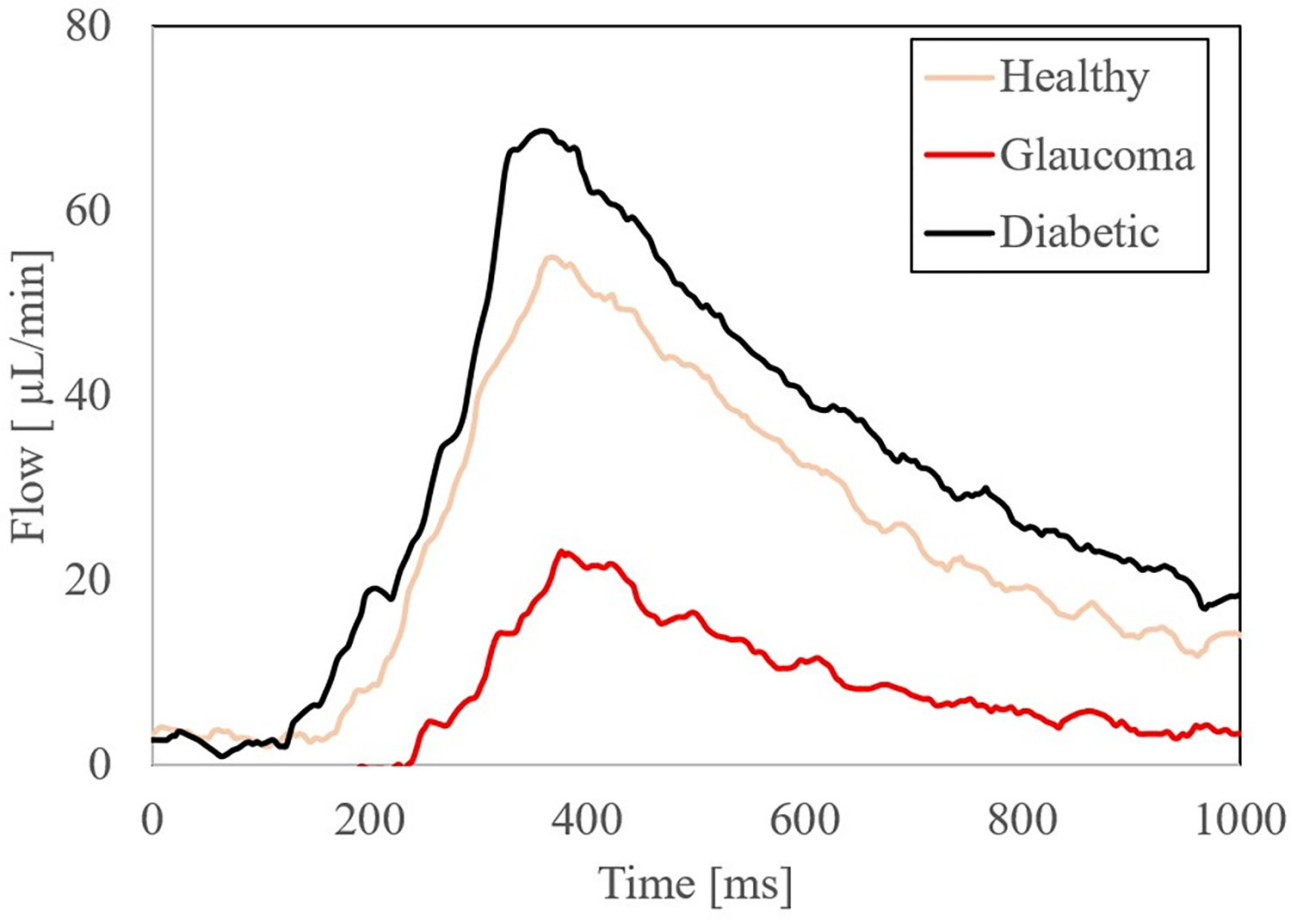
Waveform pattern simulation. Visual representation of simulation using reciprocating pump of one-second flow waveform patterns of retinal blood flow in a healthy, diabetic, and glaucoma patient scenarios generated from a computational framework.

To better illustrate the effects of input volume changes on individual pulsation amplitude and pattern, Fig 9A–9D illustrates a 1 second, single beat comparison of mean, upper and lower flow rate limits of standard deviation of measurement in ocular choroidal, total pulsatile ocular, and retinal blood flow rates. One second, single beat comparisons were not shown for organ system simulations where flow rates dropped below 0.06ml/min, waveform amplitudes exceeded the flow rate limit of the flow sensor used (65ml/min), or similarity in single beat waveforms made the subsequent graph unclear. Fig 10 illustrates the direct simulation done using the reciprocating pump of retinal blood flow rates patterns from previous literature, matching fluid rise times, peak amplitude, and total beat time similar to the Figure presented by Rebhan et. al [7].

In the simulation seen in Fig 8(B), very high flow rates (above 6ml/min) were high enough to require the use of the reciprocating action of the pump before the full 20 seconds has elapsed, requiring the pump to return to its original, fully retracted position state and explains the lack of pulsatile flow across the full 20 seconds.

## Discussion

Using the testing setup, volumetric analysis of pump output was accurate within a standard deviation range of 0.00151ml/min to 0.14196ml/min through the low (0ml/min– 0.4ml/min), mid (0.4ml/min– 1.3ml/min) and high (3ml/min– 16ml/min) bulk flow rate ranges within the organ systems tested (Fig 3). Through this, the pump has shown to provide accurate flow in ranges in which the development of mechanistic in-vitro models and products requires. Although specific organ systems were used to define the flow ranges tested, these simulations for organ systems are used as example vascular bodies and the versatility of the pump expands beyond these examples. This versatility is also created through the implementation of the reciprocating action of the pump once syringe limits have been reached. The reciprocating action of the pump takes approximately eight seconds and stops flow for that duration of time. However, the total bulk flow output, beat frequency and time input of flow are not affected.

This is one way in which the reciprocating positive displacement pump system distinguishes itself, making it ideal for use in long term fluid modeling scenarios where output variables from the model are dependent on input pulsatile flow. The use of the proprietary program along with its ability to take in user input for heart rate, volume rate and amplitude, also employs a user-input time variable where the program can calculate the exact number of pulsations within the input time frame and executing them, excluding time of retraction. This in turn, allows for precision in long term applications of the pump and makes the versality of pump applications span much wider than the organ systems described throughout this paper, although the applications within the organ systems are vast in themselves.

### Human ocular and retinal blood flow

Unlike previous pump flow systems, non-harmonic pulsatile flow can be achieved, through the manipulation of volume rate, beat rate and amplitude. This means that low volume pulsatile flow simulations are no longer limited by the method of simulating them, but rather the accuracy of the measurement method itself and its application, assuming it is within the achievable range of the pump. This can be seen through the successful simulation of multiple methods of measuring retinal and total pulsatile ocular blood flow seen in Table 1 and Fig 3. Individual pulsations utilizing our reciprocating pump are also capable of being simulated at low flow rates, through the manipulation of BPM and amplitude input. This allowed the pump to reproduce the individual pulsations of blood flow through the human retina seen in Fig 10. Previous literature has shown that flow shear stress is an important indicator of vascular disease [8, 26]. Our pump, with its ability to directly manipulate flow amplitude, a key component to shear stress in arteries, provides an avenue in which its incorporation into a flow based in-vitro model can provide comprehensive insight into the role shear stress plays in retinal disorders. The use of this system in conjunction with an integrated in-vitro model of the human eye does not need to be confined within the domain of the retina. The successful simulation of choroidal blood flow rates and waveforms makes the pump ideal to be integrated into an in-vitro model, an example an in-vitro investigation of retinal detachment. Retinal detachment has been shown to be correlated to central ocular choroidal blood flow with atrophy of the retinal pigment epithelium [27]. Because of current limitations in the simulation of low volume fluid flow models, static in-vitro models with short perfusion times are often resorted to when looking at this phenomenon [28]. The pumps implementation in in-vitro models can provide a more comprehensive understanding of the effects of blood flow on retinal detachment and other diseases. Innovations in vascular development and perfusion methods for future in-vitro models can further increase model accuracy and applicability of the pump. One early possibility can be seen through the development of optical vascular structures developed using optical coherence tomography [29]. The pulsatile nature of the fluid flow has yet to be accounted for in an in-vitro setup involving the human eye, although waveform dynamics have been shown to affect patient outcome in specific scenarios [30].

### Human fingertip and skin surface blood flow

The reciprocating positive displacement pump is also capable of simulating fingertip and skin surface blood flow under varying physiologic and pathophysiologic scenarios. An example application within this specific vascular domain is in the bioprinting of skin grafts for burn victims, where functional vascular endothelial cells require perfusion with a peristaltic pump [31]. The use of peristaltic pumps has been compared to harmonic wave flow because of its low peak-to-peak amplitude per pulse and lack of a significant flow drop between pulsations [32]. Pulsatile shear rate has been shown to increase the proliferation of vascular endothelial cells, meaning the implementation of the reciprocating positive displacement pump in a cell perfusion setup used for 3D printed cells can perhaps improve the rate and complexity at which vasculature develops, and in turn accelerate the progression towards a viable bio-printed skin-graft [9, 28]. Not only can the increased shear rates of output fluid flow affect the rate at which vascular tissue develops, but also the contents of the fluids used to perfuse the cellular structure. In our setup, the simple silicone tubing system used alongside the reciprocating positive-displacement pump allows for a vast array of fluids with physiologic or pathophysiologic makeup. An example fluid explored in the previous literature is cerebrospinal fluid (CSF), where the reciprocating positive displacement pump can be used to model pulsatile flow, shear rate, shear stress, and amplitude simultaneous to variations in CSF composition as this is directly relevant to pathologic states like those in hydrocephalus [33, 34].

### Splenic blood flow in rats

Non-human simulations using our pump are presented because of possible limitations in the collection of flow data in humans in-vivo and in turn, limitations of data for a range of organ systems. Animal models are the main alternative to in-vitro modeling; although useful in their ability to model systemic effects and device compatibility, are time-intensive to setup, limited in the range of conditions testable, and whose flow conditions may not map to humans linearly. Modeling animal flow rates gives us flexibility to eliminate unknown variables, and perhaps may reflect on human data if there are anatomical similarities between species. An example of this within this organ system domain are splenic baroreceptors and their control over splenic afferent nerve activity [35]. Novel developments in carbon-based organic semiconductors can be implemented as an afferent nerve substitute where their efficacy relative to the original nerve in a rat-based in-vitro model with pulsatile fluid flow can be modeled by the reciprocating positive displacement pump [36].

### Human cerebral arterial blood flow

The ability to isolate flow from different sources provides further versatility of the reciprocating positive displacement pump, as seen in the ophthalmic artery simulation as well as the total pulsatile ocular and retinal blood flow simulations presented in Table 1 and Fig 3. The use of anticancer chemotherapy drugs has been shown to have complications with many organs in the human body, one being the induction of ocular toxicity and other complications with the eye and retina [37, 38]. Past in vitro models of the human eye used primary human retinal endothelial cells, employing relatively short perfusion times of chemotherapeutic agents [39]. The pump can act as a method to perfuse the cells over a much longer period and can be a more comprehensive in-vitro test with more accurate flow shear rates throughout treatment. The isolated effects of a chemotherapeutic drug can also be measured, through the pumps ability to isolate specific flow rates of different vascular systems and arteries and get a much more tailored flow modelling setup. The simulation of the middle meningeal artery has fewer applications for the simulation in and of itself, although this additional application confirms that the pump is capable of simulating flow rates necessary for the study of disease not well implemented to date.

### Limitations of the reciprocating positive displacement pump

The pump is required to retract back to its initial state and reset after a set time simulating pulsatile fluid flow depending on the volume flow rate input profile used. This retraction time, and in turn pause time on the induction of fluid flow, lasts around eight seconds in the current setup. Although this is a minimal pause in fluid flow production, future iterations of the pump and proprietary program design can significantly reduce this time to near zero ensuring a constant pulsatile fluid flow is delivered to a fluid setup or model being investigated. The current syringe volume of 3ml in the pump limits the range of higher flow rate models that can be simulated, something a peristaltic pump can achieve through the sequential addition of peristaltic pump outputs in series. This can be addressed in future iterations of the pump by increasing syringe volume or using the same approach as a sequential peristaltic pump system where combining channels of the same pump and channels from multiple pumps would deliver a significantly larger volume of pulsatile fluid flow.

## Conclusion

In conclusion, the validation of the reciprocating positive displacement pump determined it was accurate in the simulation of arterial blood flow in human ocular, human fingertip and skin surface, human cerebral, and rodent spleen organ systems. This provides new opportunities in the development of novel in-vitro benchtop models involving pulsatile fluid flow and can accelerate the development of translatable treatments to improve patient outcome.

## Supporting information

S1 File (ZIP)
